# Supplementary material for: Paramedic Willingness to Report Violence Following the Introduction of a Novel, Point-of-Event Reporting Process in a Single Canadian Paramedic Service
Source: Int J Environ Res Public Health. 2024 Mar 19;21(3):363. doi: 10.3390/ijerph21030363 (PMC10970047; doi:10.3390/ijerph21030363)
Supplement: Supplementary file 1 [file ijerph-21-00363-s001.zip › ijerph-2893033-supplementary/Supplementary Materials S3 - Survey Questions.pdf]

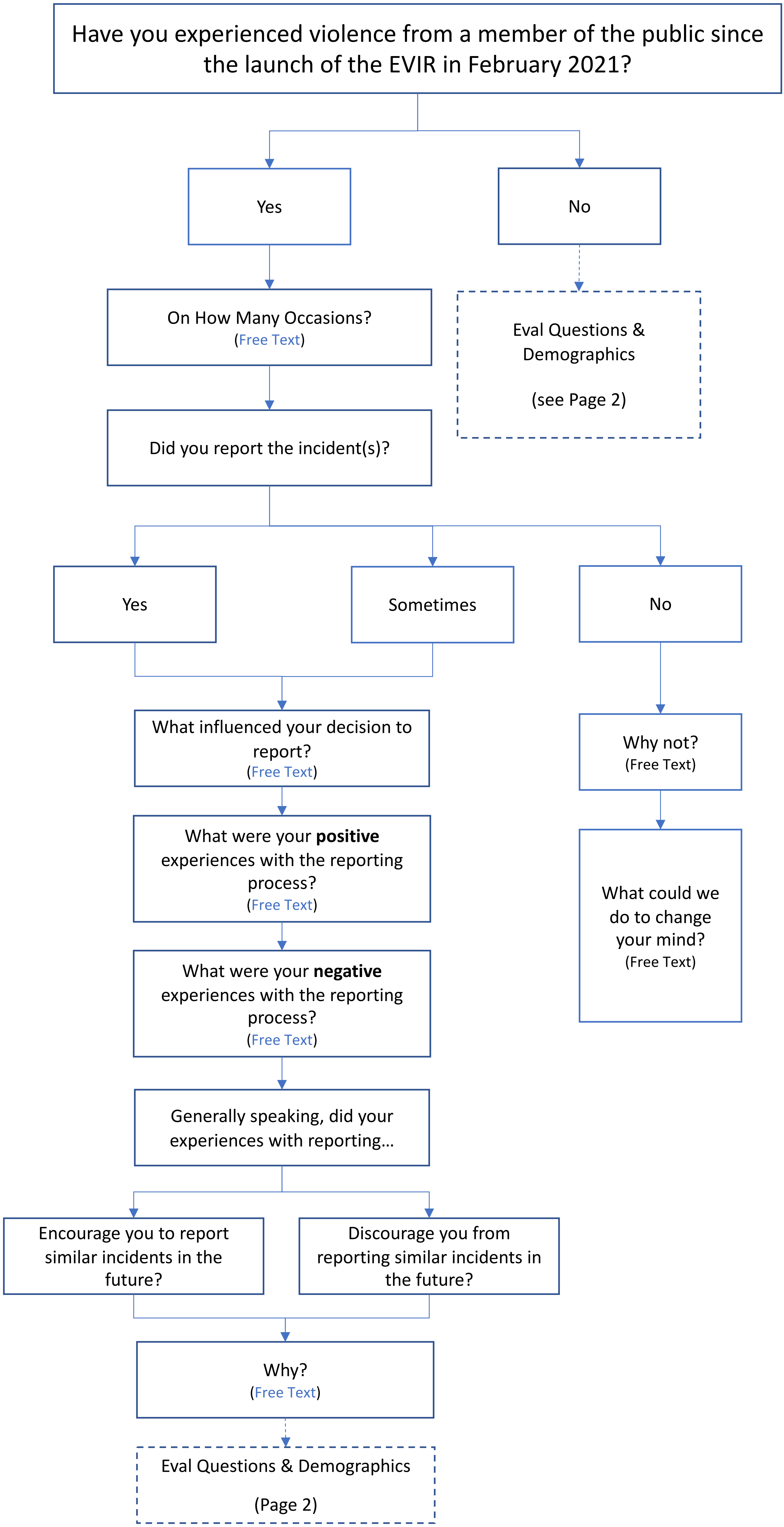
Have you experienced violence from a member of the public since the launch of the EVIR in February 2021?
Yes
No
On How Many Occasions?
(Free Text)
Eval Questions & Demographics
(see Page 2)
Did you report the incident(s)?
Yes
Sometimes
No
What influenced your decision to report?
(Free Text)
What were your **positive** experiences with the reporting process?
(Free Text)
What were your **negative** experiences with the reporting process?
(Free Text)
Generally speaking, did your experiences with reporting…
Encourage you to report similar incidents in the future?
Discourage you from reporting similar incidents in the future?
Why?
(Free Text)
Eval Questions & Demographics
(Page 2)
Why not?
(Free Text)
What could we do to change your mind?
(Free Text)

(From Page 1)

How **valuable** do you feel the following EVAP initiatives are in promoting an organizational culture within PRPS where paramedics feel **safe, supported**, and **empowered** in rejecting violence as just 'part of the job'?

EVAP Initiative	1 – Not at all Valuable	2	3	4	5 – Extremely Valuable	I Can't Say
Spit Hoods						
Soft Restraints						
Zero Tolerance Stickers in Ambulances						
SOP HS-19: Zero Tolerance for Violence from the Public Policy						
The External Violence Incident Report (EVIR)						
Address Hazard Flags Resulting from EVIRs						
Phase 1 of the Public Awareness Campaign						

(Optionally) Please use this space to provide us with any comments about the EVAP initiatives listed above. (Free Text)

What is your gender? (Man/Woman/Gender Diverse Person/Prefer Not to Say)

What is your current employment classification? (Part-Time / Full-Time [Including Temporary Full-Time Contract])

What is your current certification level? (PCP / ACP)

Are you in leadership / supervisory role? (Acting Superintendent / Superintendent / No)

For how many years have you been working as a paramedic (in total, across all paramedic services)? (Free Text)

Thank you for participating in this survey. Your feedback is important to the success of the EVAP program and helps keep paramedics in Peel Region safe.

For first responder-specific mental health support, including self-assessment tools and information on finding a mental health provider, please visit: www.pspmentalhealth.ca

For questions about this survey, please email justin.mausz@peelregion.ca
