# Supplementary figures and images for: Paramedic Willingness to Report Violence Following the Introduction of a Novel, Point-of-Event Reporting Process in a Single Canadian Paramedic Service
Source: Int J Environ Res Public Health. 2024 Mar 19;21(3):363. doi: 10.3390/ijerph21030363 (PMC10970047; doi:10.3390/ijerph21030363)

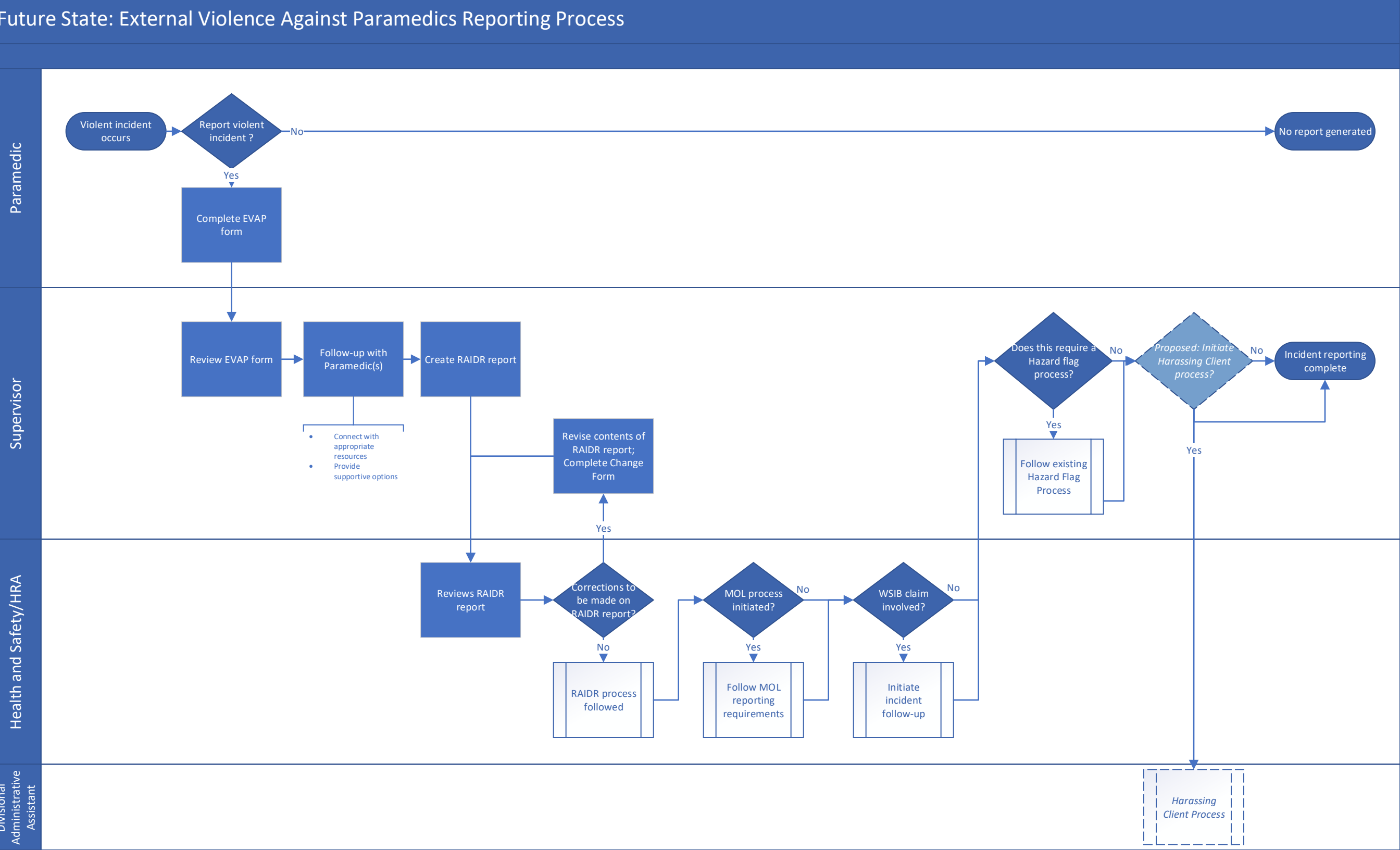

Supplement: Supplementary file 1 [file ijerph-21-00363-s001.zip › ijerph-2893033-supplementary/Supplementary Materials S2 - EVIR Actions.pdf]
